# Nonalcoholic fatty liver disease is specifically related to the risk of hepatocellular cancer but not extrahepatic malignancies

**DOI:** 10.3389/fendo.2022.1037211

**Published:** 2022-11-25

**Authors:** Somaya Albhaisi, Donna McClish, Le Kang, Tamas Gal, Arun J. Sanyal

**Affiliations:** ^1^ Department of Internal Medicine, Virginia Commonwealth University, Richmond, VA, United States; ^2^ Department of Biostatistics, Virginia Commonwealth University, Richmond, VA, United States; ^3^ Divsion of Gastroenterology, Hepatology and Nutrition, Department of Internal Medicine, Virginia Commonwealth University, Richmond, VA, United States

**Keywords:** NAFLD, NASH, cancer, obesity, diabetes mellitus, metabolic syndrome

## Abstract

**Objective:**

We performed a matched cohort study among individuals with and without nonalcoholic fatty liver disease (NAFLD) to determine: 1) the incidence of cancers (extrahepatic and liver) and their spectrum and 2) if NAFLD increases the risk of extrahepatic cancers.

**Methods:**

The NAFLD and non-NAFLD (control) cohorts were identified from electronic medical records *via* International Classification of Diseases (ICD) codes from a single center and followed from 2010 to 2019. Cohorts were matched 1:2 for age, sex, race, body mass index (BMI), and type 2 diabetes.

**Results:**

A total of 1,412 subjects were included in the analyses. There were 477 individuals with NAFLD and 935 controls (median age, 52 years; women, 54%; white vs. black: 59% vs. 38%; median BMI, 30.4 kg/m^2^; type 2 diabetes, 34%). The cancer incidence (per 100,000 person-years) was 535 vs. 1,513 (NAFLD vs. control). Liver cancer incidence (per 100,000 person-years) was 89 in the NAFLD group vs. 0 in the control group, whereas the incidence of malignancy was higher across other types of cancer in the control group vs. in the NAFLD group.

**Conclusions:**

The overall extrahepatic cancer risk in NAFLD is not increased above and beyond the risk from background risk factors such as age, race, sex, BMI, and type 2 diabetes.

## Introduction

Cancer is one of the leading causes of death in the United States and worldwide (1, 2). There is a large body of evidence that proves the association between malignancy and excess body weight (3–5). Most studies reported an increased incidence of gastrointestinal (GI) and hormone-related malignancies in individuals with obesity (4). Obesity has become a worldwide epidemic of the modern age (6, 7); therefore, the incidence of nonalcoholic fatty liver disease (NAFLD) has increased exponentially (8–10). NAFLD is closely associated with obesity and is seen in up to 80% of people with obesity (11). Less than 20% of patients with NAFLD have a normal body mass index (BMI) and no metabolic disorders (12). Numerous studies have established that malignancy is the second most frequent cause of death among patients with NAFLD (13, 14). Predictably, hepatocellular carcinoma (HCC) is the type of cancer that NAFLD is considered a major risk factor for, and this has been unanimously agreed upon by all relevant studies (15). Regarding extrahepatic malignancies, it is not known what specific types of cancer or the magnitude of risk that patients with NAFLD are at higher risk for compared to those without NAFLD. Furthermore, it remains unclear whether there are particular characteristics of malignancy risk among those with NAFLD that are distinct from those with obesity alone. A recent study by Allen et al. (16) has investigated the effect of NAFLD vs. obesity on incident cancers in a historical cohort of adults with NAFLD in Olmsted County, Minnesota, compared with age- and sex-matched controls. They reported that NAFLD and not obesity alone was associated with increased cancer risk, particularity of GI types (16). This study did not match cases and controls in BMI; instead, they used Poisson regression to examine the effect of NAFLD vs. obesity on malignancy risk. Another study by Kim et al. (17) reported that NAFLD is a risk factor for male colorectal carcinoma; however, it is important to note that they did not fully account for the interference of obesity on cancer risk (17). A study investigating the association between BMI and the development of GI cancers used BMI stratification and concluded that the NAFLD–GI cancer association was stronger in a population without obesity (18). NAFLD, like obesity, is not a localized disorder but rather a multisystem disease related to metabolism; therefore, it is highly essential to evaluate its role independently of obesity and metabolic dysregulation in certain diseases. Moreover, the question about the need for more accurate tools to characterize excess adiposity is being raised, such that BMI alone is an insufficient marker of obesity and may overlook other key contributors to disease outcomes. In order to support the importance of ruling out the effect of obesity when studying the role of NAFLD in extrahepatic malignancies, we aimed to determine the incidence and spectrum of the most common cancer types in the NAFLD population matched in age, sex, race, BMI, and type 2 diabetes with a non-NAFLD population.

## Methods

### Study population

We constructed a matched cohort study in a single center in the state of Virginia. The index dates for NAFLD cohort identification were between 2010 and 2012, and the study follow-up time was between 2010 and 2019. The two groups were identified from electronic medical records. The NAFLD cohort was composed of adults diagnosed with NAFLD. Each patient with NAFLD was individually matched to two individuals without NAFLD (control) at the time of index NAFLD diagnosis date who did not have a diagnosis of any known liver disease during the study inclusion period. Characteristics of the study population are summarized in **Table 1**. Individuals with NAFLD were identified by the International Classification of Diseases, Ninth Revision, Clinical Modification (ICD 9-CM) codes for NAFLD, which included code numbers 571.5 (cirrhosis of the liver without mention of alcohol), 571.8 (other chronic nonalcoholic liver disease), and 571.9 (unspecified chronic liver disease without mention of alcohol) and ICD-10-CM codes K75.81 [nonalcoholic steatohepatitis (NASH)] and K76.0 (fatty liver, NOS) (**Appendix Table 1**), along with elevated alanine aminotransferase (ALT) and/or aspartate aminotransferase (AST) (defined as ALT and AST ≥30 for men; ≥20 for women), radiographic evidence of hepatic steatosis, and absence of other liver diseases within 3 years prior to NAFLD diagnosis index date (**Appendix Table 1**). The control cohort was defined by absence of any known liver disease, normal liver enzymes, and hepatic imaging without fatty liver within 3 years prior to the index visit date. Those with any prior history of cancer prior to the index date or BMI ¾15 or ≥60 kg/m^2^ within 3 years prior to the NAFLD diagnosis index date were excluded for both groups. In addition, we excluded all study individuals with no healthcare visit/encounters after the index date or with less than 1 year of follow-up. The two groups were matched 1:2 for age, sex, race, BMI, and type 2 diabetes. One of the study investigators (SA) reviewed the complete medical records of a 10% random sample of individuals with NAFLD codes to confirm the validity of the code-identified study participants. In-depth chart review identified NAFLD diagnosis with a positive and negative predictive value of 86% and 87%, respectively.

**Table 1:** Baseline demographic characteristics of the study population.

	Total (n=1,412)	NAFLD (n=477)	Control (n=935)	p-value*
**Women, n (%)**	765 (54%)	269 (56%)	496 (53%)	0.2555
**Race, n (%)**				0.4892
** African American**	532 (38%)	174 (36%)	358 (38%)	
** White**	829 (59%)	274 (57%)	555 (59%)	
** Other**	51 (3%)	29 (7%)	22 (2%)	
**BMI group, n (%)**				0.2128
** 1 (<25 kg/m^2^)**	276 (19%)	85 (18%)	191 (20%)	
** 2 (25–30 kg/m^2^)**	390 (28%)	122 (26%)	268 (28%)	
** 3 (30–35 kg/m^2^)**	349 (25%)	118 (25%)	231 (25%)	
** 4 (35–40 kg/m^2^)**	232 (16%)	88 (18%)	144 (15%)	
** 5 (≥40 kg/m^2^)**	165 (12%)	64 (13%)	101 (11%)	
**BMI, median (IQR)**	30.4 (26–36)	31 (27-36.6)	30.1 (26-35.6)	0.0656
**Age, median (IQR)**	52 (44-60)	51 (43-59)	52 (44-60)	0.2728
**ALT, median (IQR)**	21 (16-33)	51 (32-84)	18 (14-21)	<0.0001
**AST, median (IQR)**	21 (17-30)	41 (28-69)	19 (16-21)	<0.0001
**Diabetes, n (%)**	473 (34%)	173 (36%)	300 (32%)	0.1153

*p-value for statistical assessment of group difference between NAFLD and control. For continuous variables, Wilcoxon signed-rank test was considered. The chi-square test was used for categorical variables. IQR, interquartile range.

### Outcomes

Both groups were followed prospectively until death, last medical visit, or December 2019. Primary outcomes were incident cancers documented after the index NAFLD diagnosis. We looked into all cancers without limitation to certain classifications or subgroups. The cancer ascertainment was done by identifying the cancer diagnoses in the medical records using the ICD-9 and ICD-10 codes documented at least once at separate dates. The cancers of interest were the most common cancers, which were classified into two groups: hepatic (liver) and extrahepatic cancers [gastrointestinal (colon, esophageal, gastric, and pancreatic), breast, uterine/endometrial, ovarian, prostate, lung, kidney/urinary tract, blood/bone marrow, and skin].

One of the study investigators (SA) reviewed the complete medical records of a 10% random sample of individuals with cancer codes to confirm the validity of the code-identified outcomes. In-depth chart review identified cancer diagnosis with a positive and negative predictive value of 87% and 88%, respectively. Comorbidities of interest included type 2 diabetes, hypertension, lipid disorders, and psoriasis. We did not have data about smoking status at the time of diagnosis or matching. Comorbidities were defined based on diagnostic ICD-9 and ICD-10 codes (**Appendix Table 2**). The study was approved by the Institutional Review Board as an institutional review board exemption under 45 CFR 46.101 (b).

### Statistical analysis

In order to reduce the confounding effects, paired matching in age, sex, race, BMI, and diabetes status was performed using propensity score matching. Baseline demographic characteristics were compared between NAFLD and matched control group using Wilcoxon signed-rank test for continuous variables (due to skewed distribution of data) and chi-square test for categorical variables. The Kaplan–Meier curves were estimated for cancer survival, along with the log-rank test for difference in survival probabilities between the NAFLD and control groups. Cancer types were identified in the electronic medical records using the codes listed in **Appendix Table 3**, and cancer incidence was estimated for both groups. The incidence rates were calculated per 100,000 person-years. Statistical analyses were performed using SAS Version 9.4 (SAS Institute, Cary, NC, USA).

## Results

**Figure 1:**
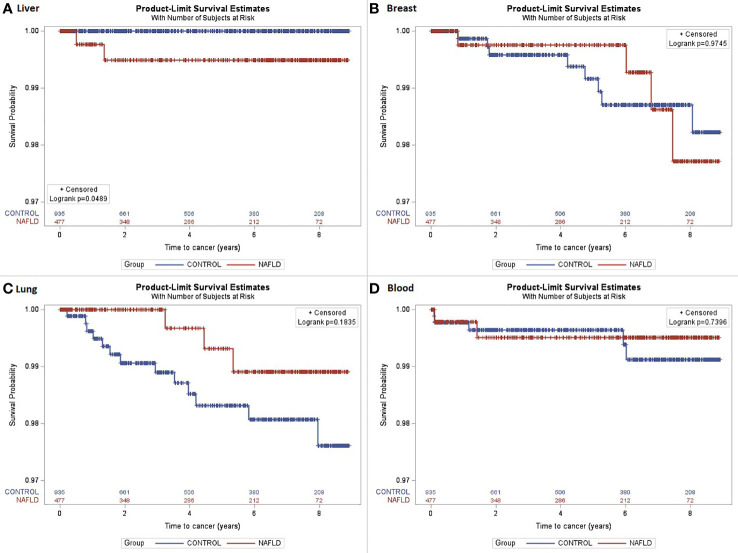
The Kaplan–Meier curves were estimated for cancer survival. **(A)** Liver cancer survival (log-rank p = 0.0489); **(B)** Breast cancer survival (log-rank p = 0.9745); **(C)** Lung cancer survival (log-rank p = 0.1835); **(D)** Blood/bone marrow cancer survival (log-rank p = 0.7396).

**Figure 2:**
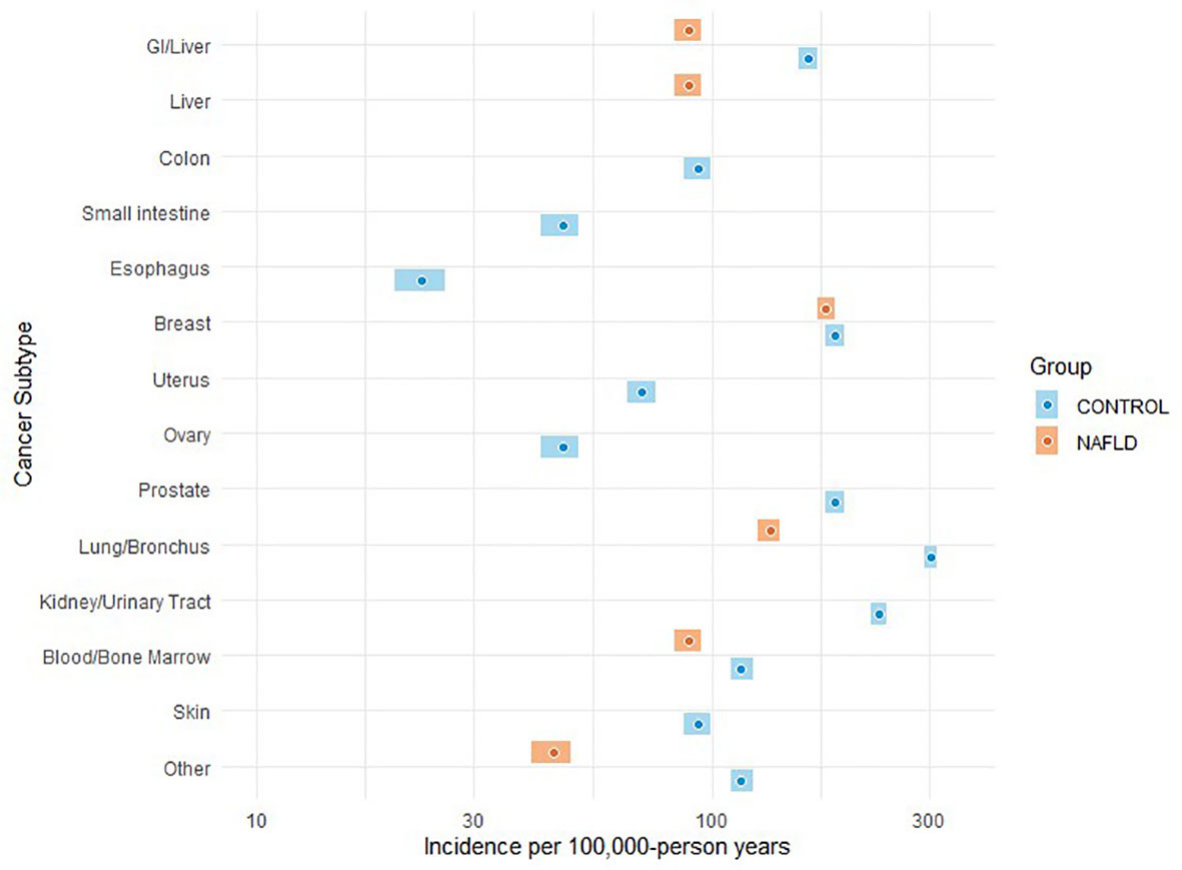
Plot chart showing the cancer incidence among individuals with NAFLD compared to age-, sex-, race-, BMI-, and diabetes-matched controls.

A total of 1,412 subjects were included in the study (NAFLD vs. control: 477 vs. 935). The median age was 52 years, with 54% of the subjects being women. The majority of subjects were Caucasian (59%) with a median BMI of 30.4 kg/m^2^. The proportion of those who have type 2 diabetes was 34%. The median follow-up was 5.7 vs. 5.2 years (NAFLD vs. control). Individuals with NAFLD had a higher proportion of obesity, i.e., BMI ≥30, compared with controls (56% vs. 51%). A total of 77 incident cancer cases (12 in the NAFLD group and 65 in the control group) were identified after matching during follow-up (total follow-up time in years: 2,244.2 for NAFLD and 4,293.8 for control). The overall cancer incidence (per 100,000 person-years) was 535 vs. 1,513 (NAFLD vs. control). More specifically, HCC incidence (per 100,000 person-years) was 89 in NAFLD vs. 0 in control, whereas the incidence of malignancy was higher across other types of cancer in control vs. NAFLD. The most common cancer in the matched control group was lung cancer as compared to breast cancer in the NAFLD group. There was no significant difference in cancer survival between NAFLD and control groups except for HCC, which was associated with higher mortality in the NAFLD group as compared to that in the control group (p = 0.0489); this was expected given that no one in the control group developed HCC. The spectrum and incidence of cancers are shown in **Table 2** and **Figures 1**, **2**.

**Table 2:** Spectrum of cancers in NAFLD and controls.

Cancer type	Cancer event count		Incidence per 100,000 person-years [with 95% confidence interval (CI)]	
	NAFLD	Control	NAFLD	Control
**Gastrointestinal/liver cancers**	2	7	89 (83, 95)	163 (155, 171)
** -Liver**	2	0	89 (83, 95)	–
** -Colon**	0	4	–	93 (87, 99)
** -Small intestine**	0	2	–	47 (42, 51)
** -Esophagus**	0	1	–	23 (20, 26)
**Breast**	4	8	178 (170, 186)	186 (178, 195)
**Uterus**	0	3	–	70 (65, 75)
**Ovary**	0	2	–	47 (42, 51)
**Prostate**	0	8	–	186 (178, 195)
**Lung/bronchus**	3	13	134 (126, 141)	303 (292, 313)
**Kidney/Urinary Tract**	0	10	–	233 (223, 242)
**Blood/Bone Marrow**	2	5	89 (83, 95)	116 (110, 123)
**Skin**	0	4	–	93 (87, 99)
**Other**	1	5	45 (40, 49)	116 (110, 123)

## Discussion

Our study highlights the importance of ruling out the effect of obesity when studying the association between NAFLD and the risk of extrahepatic complications such as malignancy. In this study, we found that the risk for extrahepatic malignancies is not increased above and beyond the risk from background risk factors that include age, sex, race, BMI, and type 2 diabetes. This finding is not in agreement with previous studies that reported that NAFLD by itself can be a risk factor for extrahepatic malignancies (16–18). However, similar to previous studies, we found that NAFLD was associated with an increased risk of HCC (15). The contradiction between our findings and what has been reported previously is quite puzzling but should not be dismissed because this brings us back to the importance of understanding the NAFLD-obesity-metabolic comorbidities conundrum. Interestingly, studies involving extrahepatic cancer risk and NAFLD, including our study, have selected different methods and statistical approaches to answer this important research question. At times, that could be one of the reasons for the differences in findings across studies, but there are numerous factors implicated. Approaching cancer prevention and management in NAFLD from the perspective of multisystem disease in the context of obesity and metabolic dysregulation is likely to be more effective in improving clinical practice and patient care than from the perspective of a single system driving cancer risk. In fact, there is currently a global multi-stakeholder endorsement of the new metabolic dysfunction-associated fatty liver disease (MAFLD) definition as an overarching term that describes fatty liver diseases associated with metabolic dysregulation because it more accurately reflects the underlying pathogenesis of the disease than does the previously used term, NAFLD, and this designation will advance the science of fatty liver disease and improve patient care (19–21). Obesity is a key driver of carcinogenesis and the development of cancers (22). The underlying mechanisms for cancer development might be attributed to metabolic dysregulations related to obesity. Several previous relevant studies did not match the study populations in BMI. Instead, they performed subgroup analyses separately for subgroups with and without obesity. This methodology, while plausible, may remain inadequate in confidently removing the confounding effect of obesity on cancer risk in NAFLD individuals. Our study provides estimates of cancer types that commonly occur in NAFLD and control groups. After matching major risk factors that include BMI, we show that NAFLD is unlikely to solely be responsible for mediating cancer risk independently of preexisting metabolic risk factors. With or without NAFLD, obesity and metabolic dysregulation remain major drivers of cancer risk. Perhaps the contribution of metabolic dysregulation to cancer risk is bigger than that of obesity because there are many obesity phenotypes that do not include fatty liver due to genetic predisposition and are not linked to increased risk of cancer (23–25). Some, but not all phenotypes, may increase the risk of cancer. Other fat distribution patterns and patterns of ectopic lipid deposition reflecting metabolic dysregulation may be linked to an elevated risk of certain types of cancer—not just excess adiposity alone. These fat distribution patterns typically reflect insulin resistance and “metabolic” obesity that includes deposition of lipid in the visceral cavity, skeletal muscle, pancreas, and kidney—these can all occur in the absence of NAFLD (26–28). A recent study (16) suggested that NAFLD was associated with a higher risk of incident cancers, while obesity alone was not; however, ectopic fat deposition, which cannot be measured by BMI, seems to be the common underlying factor in the pathogenesis of metabolic disorders and metabolic cancers (29). Instead of considering NAFLD as a mediator of the obesity–cancer association, we suggest metabolic dysregulation to be a possible mediator of this association. Measuring metabolic dysregulation is a significant barrier for studying the NAFLD-obesity-cancer relationship. Whether NAFLD directly causes cancer is difficult to establish, and there could be another proximate cause for both fatty liver and cancer (29). It is known that weight loss of 5% reverses NAFLD. However, the Women Health Initiative study indicates that while an intentional 5% weight loss reduced the risk of endometrial cancer, it did not reduce the risk of other obesity-related cancers (colon, breast, pancreas, kidney, thyroid, or liver) (29, 30). The ultimate proof will probably come from long-term follow-up of patients specifically treated for NAFLD without other metabolic disturbances; this should clarify whether the ultimate cause lies in the liver or in the adipose tissue (29). Despite previous studies suggesting the higher risk of extrahepatic cancers in patients with NAFLD, this did not result in significant changes in clinical practice; however, there is a unanimous agreement on the importance of finding reliable and cost-effective noninvasive diagnostic markers of NAFLD to improve clinical care and facilitate NAFLD research. There is strong evidence to support the positive association between obesity and most common cancers (4, 31). Proposed mechanisms for this association include insulin and other hormones, insulin-like growth factor 1, adipokines, and systemic inflammation (32–34). Diabetes mellitus is another important confounding factor for the NAFLD–cancer association given that insulin resistance is a plausible mechanism linking cancer with NAFLD, which is why we matched the groups in type 2 diabetes as well. However, it cannot be assumed that matching diabetes appropriately accounts for differences in insulin resistance because type 2 diabetes may or may not account for varying degrees of insulin resistance. In the absence of liver biopsy in the general population and formally approved noninvasive diagnostic methods in addition to the unreliability of liver enzymes as biomarkers for NAFLD, it is difficult to ascertain possible distinct associations between the different stages of NAFLD (simple steatosis, NASH) and extrahepatic malignancies. For all of the abovementioned reasons, the remote effects of NAFLD leading to extrahepatic malignancies remain unclear. Our findings can be applied in clinical practice to guide counseling in individuals with obesity and to support larger studies investigating the effectiveness of cancer screening in obesity. This study has major limitations, which include the usual potential sources of bias seen with observational studies, sample size, duration of follow-up, being a single-center study, lack of reliable biomarkers for the diagnosis of NAFLD, unavailability of liver biopsy results, and unknown smoking status of both groups. The use of ICD codes to identify cases of NAFLD, which is likely to be a gross underestimate of the extent of the problem, is a major limitation of most NAFLD studies. Majority of individuals in the control group had missing lab values, so we could not estimate markers such as fibrosis-4 (FIB-4) score for comparison with the NAFLD group to evaluate for possible predictive ability of cancer risk. Furthermore, a proportion of the control group may have undiagnosed NAFLD, and we tried to address that by ensuring that they never had any ICD codes for NAFLD over the entire follow-up period; however, this method alone does not exclude possible undiagnosed or subclinical disease in the control group. Information about therapeutic interventions for obesity or NAFLD (e.g., dietary interventions, weight loss medications, etc.) and changes in BMI over time is missing in our study. The strengths of this study include the use of a cohort without NAFLD individually matched by age, sex, race, BMI, and type 2 diabetes with the NAFLD cohort. We conducted random in-depth chart review to confirm the diagnosis of cancer for both groups. We have randomly selected our study population, so it is difficult to explain why the patients were relatively young compared to previous studies, but this might be reflective of the characteristics of the patient population at our institution. This might partially explain the very low number of patients who developed HCC in addition to other factors such as underdiagnosing cancer and lack of data on regular cancer screenings. Nonetheless, the other demographic characteristics of the study population are roughly similar in general to other populations in the southern region of the United States, but any differences in the demographic distributions of other populations in other regions should be considered when attempting to generalize the results. There is no doubt that NAFLD is a multisystem disease with a vast range of complications, both intrahepatic and extrahepatic, but the more important and extremely dangerous player in driving carcinogenesis, regardless of NAFLD, is adiposity. Measures of obesity such as BMI are insufficient to accurately characterize excess adiposity given that BMI indicates neither the percentage of body fat mass nor the location of the fat (35) and would miss identification of visceral obesity even with a normal BMI can increase the risk of various extrahepatic complications in individuals with “lean NAFLD.” Our findings highlight the importance of early management of obesity and provide a rationale for future larger studies on the effectiveness of cancer screening in obesity and larger studies of NAFLD after ruling out the effects of obesity and metabolic comorbidities such as diabetes. While the results of our study did not provide new aspects to the current understanding of NAFLD as an HCC cancer risk, they will potentially bring back the debate regarding NAFLD being an independent risk factor for extrahepatic malignancies.

## Conclusion

The overall cancer risk in NAFLD is not increased above and beyond the risk from background risk factors such as age, race, BMI, and type 2 diabetes. The risk of HCC is specifically related to NAFLD in this study population. However, our conclusions are limited by major study limitations; therefore, future studies investigating the association between NAFLD and extrahepatic malignancy should account for metabolic dysregulation and its comorbidities and the possible interference of obesity in the cancer risk and should try to minimize the confounding effect of obesity. In addition, there are numerous other factors implicated in the obesity–cancer relationship, such as insulin resistance and gut microbiota, and should be considered in future studies. Our study is calling for rethinking the NAFLD–extrahepatic cancer association and for considering a holistic approach for understanding and managing metabolic comorbidities for cancer prevention.

## Data availability statement

The raw data supporting the conclusions of this article will be made available by the authors, without undue reservation.

## Ethics statement

The studies involving human participants were reviewed and approved by Institutional Review Board at Virginia Commonwealth University. Written informed consent for participation was not required for this study in accordance with the national legislation and the institutional requirements.

## Author contributions

Conceptualization, SA and AJS; Data curation, SA, DM and TG; Formal analysis, SA, DM, and LK; Investigation, AJS; Methodology, SA, DM, LK and AJS; Project administration, SA, TG and AJS; Resources, AJS; Software, DM and LK; Supervision, AJS; Validation, SA, DM and LK; Visualization, DM and LK; Writing – original draft, SA; Writing – review & editing, SA, DM, LK, TG and AJS. All authors contributed to the article and approved the submitted version.

## Funding

Services and products in support of the research project were generated by the VCU Massey Cancer Center Bioinformatics Shared Resource, supported, in part, with funding from NIH-NCI Cancer Center Support Grant P30 CA016059.

## Acknowledgments

We thank Nevena Skoro, MPH, Director of analytics – VCU Massey Cancer Center, for assisting with extracting the data and creating the database.

## Conflict of interest

AJS is President of Sanyal Biotechnology and has stock options in Genfit, Akarna, Tiziana, Indalo, Durect, Exhalenz and Hemoshear. He has served as a consultant to Astra Zeneca, Nitto Denko, Ardelyx, Conatus, Nimbus, Amarin, Salix, Tobira, Takeda, Fibrogen, Jannsen, Gilead, Lilly, Poxel, Artham, Cymabay, Boehringer Ingelhiem, Novo Nordisk, Birdrock, Novartis, Pfizer, Jannsen and Genfit. He has been an unpaid consultant to Intercept, Echosens, Immuron, Galectin, Fractyl, Syntlogic, Affimune, Chemomab, Nordic Bioscience and Bristol Myers Squibb. His institution has received grant support from Gilead, Salix, Tobira, Bristol Myers, Shire, Intercept, Merck, Astra Zeneca, Malinckrodt, Cumberland and Novartis. He receives royalties from Elsevier and UptoDate.

The remaining authors declare that the research was conducted in the absence of any commercial or financial relationships that could be construed as a potential conflict of interest.

## Publisher’s note

All claims expressed in this article are solely those of the authors and do not necessarily represent those of their affiliated organizations, or those of the publisher, the editors and the reviewers. Any product that may be evaluated in this article, or claim that may be made by its manufacturer, is not guaranteed or endorsed by the publisher.
