# Supplementary material for: Nonalcoholic fatty liver disease is specifically related to the risk of hepatocellular cancer but not extrahepatic malignancies
Source: Front Endocrinol (Lausanne). 2022 Nov 25;13:1037211. doi: 10.3389/fendo.2022.1037211 (PMC9732089; doi:10.3389/fendo.2022.1037211)
Supplement: Supplementary file 1 [file DataSheet_1.docx]

**APPENDIX**

**Appendix Table 1: ICD9 and 10 codes for liver diagnoses (for inclusion and exclusion)**

| **ICD-9 Codes:**  **(Liver Diseases)**  570 Acute and subacute necrosis of liver  571 Chronic liver disease and cirrhosis: 571.0, 571.1, 571.2, 571.3, 571.4, 571.40, 571.41, 571.42, 571.49, 571.5, 571.6, 571.8, 571.9  572 Liver abscess and sequelae of chronic liver disease: 572.0, 572.1, 572.2, 572.3, 572.4, 572.8  573 Other disorders of liver: 573.0, 573.1, 573.2, 573.3, 573.4, 573.5, 573.8, 573.9 |
| --- |
| **ICD-10 Codes:**  **(Liver Diseases)**  K70 Alcoholic liver disease: K70.0, K70.1, K70.2, K70.3, K70.4, K70.9  K71 Toxic liver disease: K71.0, K71.1, K71.2, K71.3, K71.4, K71.5, K71.6, K71.7, K71.8, K71.9  K72 Hepatic failure, not elsewhere classified: K72.0, K72.1, K72.9  K73 Chronic hepatitis, not elsewhere classified: K73.0, K73.1, K73.2, K73.8, K73.9  K74 Fibrosis and cirrhosis of liver: K74.0, K74.1, K74.2, K74.3, K74.4, K74.5, K74.6  K75 Other inflammatory liver diseases: K75.0, K75.1, K75.2, K75.3, K75.4, K75.8, K75.9  K76 Other diseases of liver: K76.0, K76.1, K76.2, K76.3, K76.4, K76.5, K76.6, K76.7, K76.8, K76.9  K77 Liver disorders in diseases classified elsewhere: K77.0, K77.8  B15 Acute hepatitis A: B15.0, B15.9  B16 Acute hepatitis B: B16.0, B16.1, B16.2, B16.9  B17 Other acute viral hepatitis: B17.0, B17.1, B17.2, B17.8, B17.9  B18 Chronic viral hepatitis: B18.0, B18.1, B18.2, B18.8, B18.9  B19 Unspecified viral hepatitis: B19.0, B19.9 |

**Appendix Table 2: ICD9 and 10 codes for comorbidities**

| **ICD-9 Codes for co-morbidities:**  250 Diabetes mellitus  401 Essential hypertension  272 Disorders of lipoid metabolism  696 Psoriasis and similar disorders |
| --- |
| **ICD-10 Codes for co-morbidities:**  E10-E14 Diabetes mellitus  I10-I15 Hypertensive diseases  E78 Disorders of lipoprotein metabolism and other lipidaemias  L40 Psoriasis |

**Appendix Table 3: ICD 9 and 10 codes for cancers**

| **Cancer type** | **ICD 9** | **ICD 10** |
| --- | --- | --- |
| **Breast** | 174  174.0  174.1  174.2  174.3  174.4  174.5  174.6  174.8  174.9 | C50.011  C50.012  C50.019  C50.111  C50.112  C50.119  C50.211  C50.212  C50.219  C50.311  C50.312  C50.319  C50.411  C50.412  C50.419  C50.511  C50.512  C50.519  C50.611  C50.612  C50.619  C50.811  C50.812  C50.819  C50.911  C50.912  C50.919 |
| **Esophagus** | 150  150.0  150.1  150.2  150.3  150.4  150.5  150.8  150.9 | C15.3  C15.4  C15.5  C15.8  C15.9 |
| **Stomach** | 151  151.0  151.1  151.2  151.3  151.4  151.5  151.6  151.8  151.9 | C16.0  C16.1  C16.2  C16.3  C16.4  C16.5  C16.6  C16.8  C16.9 |
| **Colon, rectum, anus** | 153  153.0  153.1  153.2  153.3  153.4  153.5  153.6  153.7  153.8  153.9  154  154.0  154.1  154.2  154.3  154.8 | C18.0  C18.1  C18.2  C18.3  C18.4  C18.5  C18.6  C18.7  C18.8  C18.9  C19  C20  C21.0  C21.1  C21.2  C21.8 |
| **Liver and intrahepatic duct** | 155  155.0  155.1 | C22.0  C22.1 |
| **Pancreas** | 157  157.0  157.1  157.2  157.3  157.4  157.8  157.9 | C25.0  C25.1  C25.2  C25.3  C25.4  C25.7  C25.8  C25.9 |
| **Bronchus and Lung** | 162.2  162.3  162.4  162.5  162.8  162.9 | C34.00  C34.01  C34.02  C34.10  C34.11  C34.12  C34.2  C34.30  C34.31  C34.32  C34.80  C34.81  C34.82  C34.90  C34.91  C34.92 |
| **Ovarian** | 183  183.0 | C56.1  C56.2  C56.9 |
| **Kidney/Urinary Tract** | 189.9 | C68.9 |
| **Prostate** | 185 | C61 |
| **Uterus, Endometrium** | 179  182  182.0  182.1  182.8 | C54.0  C54.1  C54.2  C54.3  C54.8  C54.9  C55 |
| **Blood/Bone Marrow** | 200-209 | C81-C96 |
| **Skin** | 172-176  184 | C43-44 |
